# Late‐Onset Oral Cenesthopathy With Dopaminergic Dysfunction: Therapeutic Response to Pramipexole in a Case With Suspected Prodromal Lewy Body Disease

**DOI:** 10.1111/psyg.70088

**Published:** 2025-08-27

**Authors:** Hitomi Matsui, Takehiro Tamura, Masashi Kameyama, Yuki Omori, Genichi Sugihara, Takashi Takeuchi, Hidehiko Takahashi, Ko Furuta

**Affiliations:** ^1^ Tokyo Metropolitan Institute for Geriatrics and Gerontology Itabashi Japan; ^2^ Department of Psychiatry and Behavioral Sciences, Graduate School of Medical and Dental Sciences Institute of Science Tokyo Japan; ^3^ Center for Brain Integration Research Institute of Science Tokyo Japan

**Keywords:** dopaminergic dysfunction, late‐onset depression, oral cenesthopathy, pramipexole, prodromal Lewy body disease

## Abstract

An 85‐year‐old woman with late‐onset depression subsequently developed persistent oral cenesthopathy. As antidepressant augmentation, low‐dose aripiprazole improved both mood and oral symptoms, but oversedation and parkinsonism necessitated tapering and discontinuation. After discontinuation, oral cenesthopathy recurred without clear depressive worsening. Dopamine transporter single‐photon emission computed tomography showed reduced bilateral striatal uptake and ^123^I‐metaiodobenzylguanidine cardiac scintigraphy showed decreased uptake, with preserved cognition. In light of the clinical and imaging findings suggestive of dopaminergic dysfunction, and after an informed discussion of off‐label use, pramipexole 0.25 mg daily was initiated. Oral discomfort lessened within 1 week and oral intake normalised by 2 weeks, without adverse effects; remission of oral symptoms and mood persisted for 6 months. Overall, the presentation was conceptually compatible with the psychiatric‐onset phenotype described in the prodromal Lewy body disease research framework, but this remains an interpretive consideration rather than a diagnosis. Assessing possible dopaminergic involvement in similar cases of oral cenesthopathy may aid individualised management; however, the therapeutic role of dopamine agonists requires confirmation in prospective studies.

## 1 Introduction

Cenesthopathy is a phenomenological concept in which patients persistently report bizarre and abnormal bodily sensations in the absence of identifiable physical abnormalities [1]. First described in 1907 by Dupré and Camus as a ‘localised distortion of bodily awareness’, cenesthopathy has since been divided into various subtypes. One such subtype is oral cenesthopathy, which is characterised by persistent symptoms, such as a foreign body sensation in the mouth or a burning sensation in the absence of observable clinical signs or structural abnormalities. In Japan, while non‐oral forms of cenesthopathy are more common in young men, oral cenesthopathy predominantly affects middle‐aged and older women aged 40 and above, and cases in this age group are classified in this report as late‐onset [1, 2].

Oral cenesthopathy is not recognised as an independent diagnostic entity in the current edition of the Diagnostic and Statistical Manual of Mental Disorders (DSM‐5‐TR). In clinical settings, it is often interpreted as a psychotic symptom, such as somatic delusion or hallucination, and is therefore commonly treated with antipsychotic medications. However, no standardised or consistently effective treatment for oral cenesthopathy has been established. This diagnostic and therapeutic ambiguity likely reflects the limited understanding of the pathophysiological basis of this phenomenological concept.

Recent studies have suggested that dopaminergic dysfunction is involved in oral cenesthopathy [3, 4]. In our previous case series, dopamine transporter single‐photon emission computed tomography (DaT‐SPECT) with ^123^I‐ioflupane showed reduced striatal uptake in 11 of 12 patients (92%), indicating impaired dopaminergic function [3]. Moreover, several clinical and neuroimaging studies have reported that certain cases of late‐onset depression and psychosis are associated with the prodromal pathology of Lewy body disease (LBD), including Parkinson's disease (PD) and dementia with Lewy bodies (DLB), with dopaminergic dysfunction considered to be a common underlying mechanism [5]. In this context—given that dopaminergic dysfunction is already implicated in prodromal LBD—oral cenesthopathy may also represent a prodromal LBD manifestation. However, to date, no studies have investigated therapeutic approaches based on this hypothesis.

This report presents a case of an elderly woman with late‐onset oral cenesthopathy and depression, raising the possibility of underlying LBD pathology; her oral symptoms improved after the initiation of the dopamine agonist pramipexole. Written informed consent for publication was obtained from the patient and her son after explaining the purpose, content and privacy protections.

## 2 Case Presentation

An 85‐year‐old Japanese woman living alone in Tokyo first developed insomnia 8 years earlier, which responded well to etizolam (0.5 mg). Three years later, she was hospitalised for heart failure and underwent aortic valve replacement. Postoperatively, insomnia recurred and was accompanied by persistently depressed mood, diminished motivation, impaired concentration, fatigue, anorexia, dizziness, head heaviness and oral discomfort. Six months after discharge, she was diagnosed with moderate major depressive disorder. Mirtazapine (7.5 mg) improved her mood and alleviated somatic symptoms.

**FIGURE 1:**
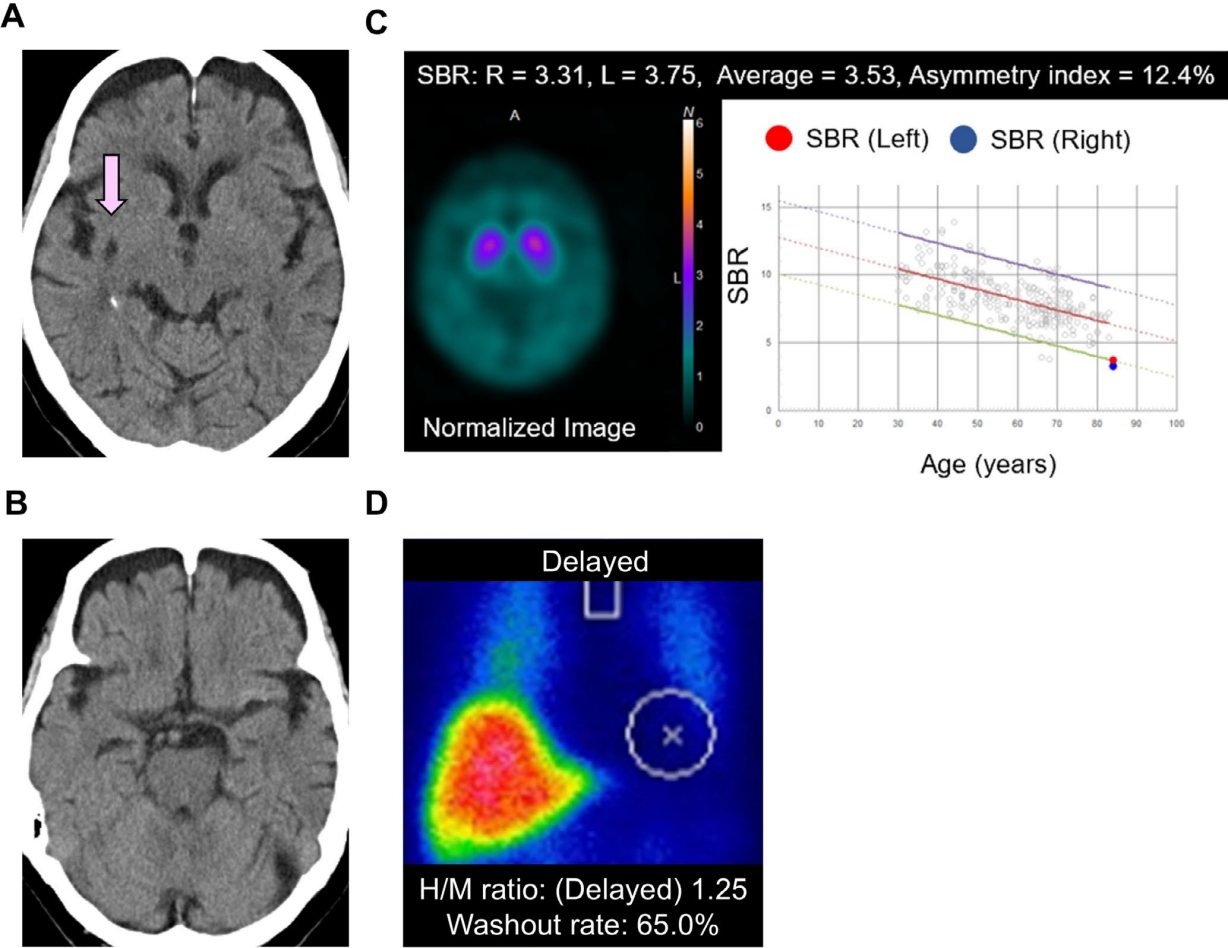
Multimodal brain and cardiac imaging in a case of late‐onset oral cenesthopathy. (A) Head CT shows signs of an old haemorrhage in the right putamen, indicated by a pink arrow. (B) Mild atrophy is observed in the frontal lobes and medial temporal regions, but no clearly pathological or age‐inappropriate atrophy is evident. (C) DaT‐SPECT using ^123^I‐ioflupane shows reduced bilateral striatal uptake (SBR: right, 3.78; left, 4.33; mean, 4.05; asymmetry index, 13.6%), with the mean value falling below the −2 SD threshold and both sides approaching or exceeding this cutoff compared with age‐matched controls, as presented in the B inset. Coloured reference lines in the inset indicate the age‐matched mean (red), +2 SD (purple), and −2 SD (green). (D) ^123^I‐MIBG cardiac scintigraphy shows reduced cardiac uptake (H/M ratio: 1.57 [early], 1.25 [delayed]; washout rate: 65%). For reference, H/M ratios > 2.20 (early and delayed) and washout rates < 34% are considered to be within the normal range.

Approximately 3 years later, she discontinued mirtazapine, and the depressive symptoms recurred. Resumption of mirtazapine partially improved her mood, but dizziness, head heaviness and oral cenesthopathic symptoms—characterised by stickiness and dysgeusia—persisted and significantly impaired her oral intake. The dose of mirtazapine was increased to 22.5 mg, but her depressive symptoms did not improve and dizziness slightly worsened, prompting her first psychiatric hospitalisation. Laboratory tests were unremarkable, and head computed tomography revealed signs of an old haemorrhage in the right putamen, with no clearly pathological or age‐inappropriate atrophy, including in the medial temporal lobes (Figure 1A,B). Aripiprazole (1.5 mg) was added to mirtazapine (15 mg) as an augmentation strategy, with reported improvement in depressive symptoms and oral cenesthopathy. However, it caused marked sedation that interfered with daily functioning. Reduction of the aripiprazole dose to 0.5 mg improved the sedation; however, dizziness and head heaviness persisted.

Based on the combination of late‐onset depression, oral cenesthopathy, persistent somatic symptoms suggestive of autonomic dysfunction (such as dizziness and head heaviness), and marked sensitivity to antipsychotic agents, we considered that the clinical picture could align with the psychiatric‐onset phenotype proposed in the prodromal LBD research framework by McKeith et al. [6] While parkinsonian motor symptoms and other core clinical features of DLB (such as visual hallucinations or rapid eye movement sleep behaviour disorder) were absent and cognitive function was relatively preserved (Mini‐Mental State Examination [MMSE] score: 28/30, with one‐point losses on delayed recall and the three‐step command), there was no evidence of subjective or objective cognitive decline. DaT‐SPECT showed reduced bilateral striatal uptake (SBR: right, 3.78; left, 4.33; mean, 4.05; asymmetry index, 13.6%) (Figure 1C). While the right‐sided reduction may reflect the prior putaminal haemorrhage, the concurrent reduction on the left may indicate dopaminergic dysfunction potentially associated with LBD. In addition to quantitative assessment, visual interpretation was performed by a board‐certified nuclear medicine specialist (Dr. M. Kameyama), who confirmed the scan as positive for striatal dopaminergic deficit. Importantly, she was not taking any medications known to affect DaT‐SPECT.

Over the next year, progressive unsteadiness, dizziness, head heaviness, and fatigue developed, accompanied by extrapyramidal symptoms, such as bradykinesia and a forward‐leaning posture, ultimately leading to rehospitalization. Aripiprazole discontinuation improved extrapyramidal symptoms but led to the recurrence of oral cenesthopathy, which impaired food intake. Given the previously identified striatal DaT reduction, we planned further management with a focus on dopaminergic mechanisms. Considering the absence of dementia‐level cognitive impairment and the risk of exacerbating parkinsonism, cholinesterase inhibitors were not considered appropriate. In light of the clinical and imaging findings suggestive of dopaminergic dysfunction, pramipexole (0.25 mg), a dopamine agonist, was initiated to address the presumed deficit; it has also shown efficacy as an augmentation agent in randomised controlled trials for depression—was initiated after careful discussion of its off‐label use with the patient and their family [7]. Within 1 week, oral discomfort diminished and solid food intake resumed; oral intake returned to near‐normal within 2 weeks. No adverse effects were observed.

During hospitalisation, prominent autonomic dysfunction was suspected, which was supported by longstanding somatic symptoms, such as dizziness and head heaviness, along with objective findings. Orthostatic hypotension—defined as a > 20 mmHg drop in systolic blood pressure—was observed. Although the patient had a history of cardiac surgery, current cardiac dysfunction was not evident. Nevertheless, ^123^I‐metaiodobenzylguanidine (MIBG) scintigraphy revealed reduced cardiac uptake (heart‐to‐mediastinum ratio: early 1.57, delayed 1.25; washout rate: 65%) (Figure 1D). Of note, she was not taking any medications, nor did she have comorbid conditions known to affect MIBG cardiac scintigraphy.

Following improvement in depressive symptoms, the patient scored 27 of 30 on the MMSE, with one point lost on delayed recall and two points lost on serial sevens. Despite these minor deficits, there was no evidence of subjective or objective cognitive impairment, and the patient did not meet the criteria for mild cognitive impairment (MCI). Based on the combination of clinical features and functional imaging studies, underlying LBD pathology was suspected; however, the patient did not meet the clinical diagnostic criteria for either PD or DLB. After stabilisation, she was discharged. At the 6‐month follow‐up, she remained free from both oral cenesthopathy and depressive symptoms and showed no parkinsonian features during the follow‐up period (Figure S1).

## 3 Discussion

This report presents a case of an elderly patient with late‐onset oral cenesthopathy and depression, in whom dopaminergic dysfunction was suspected based on reduced striatal uptake on DaT‐SPECT and the emergence of parkinsonian symptoms following even low‐dose antipsychotic exposure. Initiation of dopaminergic therapy with pramipexole was followed by marked improvement in the oral symptoms, which appeared unrelated to the course of depressive symptoms. These findings suggest that pramipexole may be beneficial in elderly psychiatric patients with refractory oral cenesthopathy and suspected dopaminergic dysfunction, possibly related to LBD.

The treatment of oral cenesthopathy remains clinically challenging. Various pharmacological and non‐pharmacological interventions—such as antidepressants, atypical antipsychotics, lithium, donepezil, electroconvulsive therapy, and psychotherapy—have been reported to provide partial or temporary benefit [1, 8, 9]. However, overall treatment response rates remain low, with estimates generally below 50% [1]. This limited effectiveness, combined with the diversity of attempted treatments, underscores the need for more individualised and pathophysiologically informed therapeutic approaches. Consequently, other mechanistic approaches—such as dopaminergic augmentation—have begun to attract interest, although systematic evidence is still sparse [4].

In the present case, oral cenesthopathy markedly improved after the initiation of pramipexole, a dopamine agonist. A prior trial of low‐dose aripiprazole provided transient benefit before parkinsonian adverse effects necessitated discontinuation. While dopamine agonists have rarely been utilised to treat oral cenesthopathy, pramipexole has been reported to alleviate not only depressive symptoms but also oral discomfort in patients with PD, a clinical subtype of LBD [4]. These reports, along with our findings, indicate that dopaminergic agonists may be a rational treatment option for oral cenesthopathy in cases where dopaminergic dysfunction is clinically suspected and supported by imaging biomarkers, even in the absence of a formal PD or DLB diagnosis. Comparable improvement with levodopa has not yet been reported, and head‐to‐head comparisons of dopamine agonists are lacking. Further controlled studies are therefore required before recommending one dopaminergic agent over another.

The mechanism by which dopaminergic stimulation ameliorates cenesthopathy remains uncertain. One hypothesis involves D3‐rich limbic circuits (e.g., nucleus accumbens) that integrate reward and interoceptive signals [10]. Low‐dose aripiprazole—a partial D2/D3 agonist—provided transient relief but induced parkinsonism, whereas pramipexole—a dopamine agonist with high affinity for D3 receptors—produced rapid, sustained improvement. Oral cenesthopathy can be viewed as a somatic hallucination linked to impaired sensorimotor gating. Prepulse inhibition (PPI) is regarded as a surrogate marker for sensorimotor gating; impaired gating has been linked to somatic hallucinations. PPI studies show that pramipexole enhances gating via D3 activation [11], and fMRI implicates prefrontal–striatal–pontine loops in this process [12, 13]. Pramipexole‐enhanced PPI therefore offers a plausible mechanism whereby D3 stimulation mitigates abnormal oral sensations. However, evidence remains limited; larger studies are needed to determine whether pramipexole's effect is chiefly dopaminergic, antidepressant or due to other mechanisms.

Regardless, in clinical cases like this one, the limited efficacy and poor tolerability of antipsychotic agents may reflect disease‐specific neurochemical vulnerabilities, such as increased sensitivity to antipsychotic‐induced dopaminergic blockade. The identification of this subgroup through clinical features or imaging biomarker‐based assessment may allow for the anticipation of adverse reactions. This, in turn, could support the development of more individualised pharmacological strategies—avoiding unnecessary antipsychotic exposure and improving symptom control, tolerability and overall clinical patient outcomes.

This report is subject to several limitations. First, as a single‐case observation, generalizability is inherently limited. Second, although the patient's response to pramipexole and imaging biomarker findings indicate dopaminergic dysfunction, causality cannot be definitively established. Third, the diagnosis of psychiatric‐onset LBD remains a research construct, and further refinement of its clinical criteria is warranted. Fourth, although a small chronic haemorrhage was observed in the right putamen, which could theoretically affect striatal dopamine uptake, the concurrent reduction in cardiac MIBG uptake reinforces the interpretation that the Lewy body pathology was present. Nevertheless, the possibility of mixed pathology, including vascular contributions, cannot be entirely excluded. Fifth, the precise mechanisms by which dopaminergic dysfunction might give rise to cenesthopathic symptoms remain unclear. Future studies should aim to systematically accumulate and characterise biomarker‐supported cases of oral cenesthopathy and evaluate responses to dopaminergic agents in elderly patients with suspected LBD pathology.

## 4 Conclusion

This case illustrates the potential utility of pramipexole in treating late‐onset oral cenesthopathy in the context of suspected dopaminergic dysfunction. Careful assessment for possible dopaminergic involvement may be informative in patients with oral cenesthopathy showing poor response or limited tolerability to antipsychotics. The accumulation of additional cases—ideally including both clinical and biomarker‐based assessments—and prospective evaluation of dopaminergic therapies are warranted to elucidate their therapeutic relevance in this population.

## Disclosure

The authors have nothing to report.

## Ethics Statement

The Ethics Review Committee at the Tokyo Metropolitan Institute for Geriatrics and Gerontology unequivocally asserts that, in principle, case reports necessitate no ethics review if individual consent is secured; thus, ethical scrutiny for this case study was exempted.

## Conflicts of Interest

The authors declare no conflicts of interest.

## Supporting information


**Figure S1:** Timeline illustrating the clinical progression of depressive and cenesthopathic symptoms, pharmacological interventions, imaging assessments, and subsequent therapeutic response to pramipexole. CT, computed tomography; DaT‐SPECT, dopamine transporter single‐photon emission computed tomography; SBR, specific binding ratio; SD, standard deviation; MIBG, metaiodobenzylguanidine; H/M, heart‐to‐mediastinum ratio.

## Data Availability

The data that support the findings of this study are available from the corresponding author upon reasonable request.
