# Supplementary figures and images for: Late‐Onset Oral Cenesthopathy With Dopaminergic Dysfunction: Therapeutic Response to Pramipexole in a Case With Suspected Prodromal Lewy Body Disease
Source: Psychogeriatrics. 2025 Aug 27;25(5):e70088. doi: 10.1111/psyg.70088 (PMC12381603; doi:10.1111/psyg.70088)

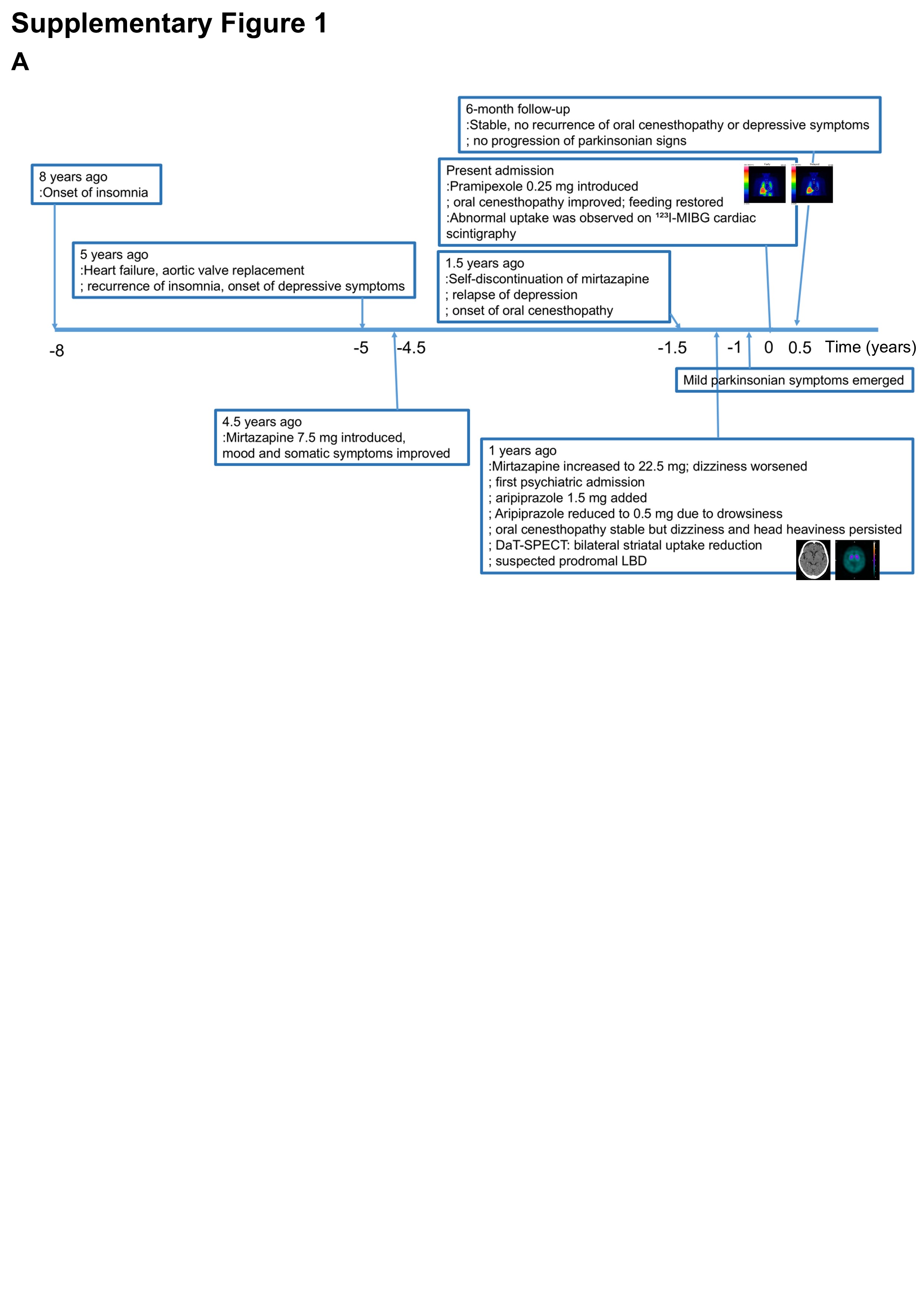

Supplement: Supplementary file 1 — Figure S1: Timeline illustrating the clinical progression of depressive and cenesthopathic symptoms, pharmacological interventions, imaging assessments, and subsequent therapeutic response to pramipexole. CT, computed tomography; DaT‐SPECT, dopamine transporter single‐photon emission computed tomography; SBR, specific binding ratio; SD, standard deviation; MIBG, metaiodobenzylguanidine; H/M, heart‐to‐mediastinum ratio. [file PSYG-25-0-s001.jpg]
